# Supplementary material for: New Insights Into Sperm Ultrastructure Through Enhanced Scanning Electron Microscopy
Source: Front Cell Dev Biol. 2021 Apr 22;9:672592. doi: 10.3389/fcell.2021.672592 (PMC8100687; doi:10.3389/fcell.2021.672592)
Supplement: Supplementary file 3 [file Data_Sheet_1.docx]

Supplementary Material


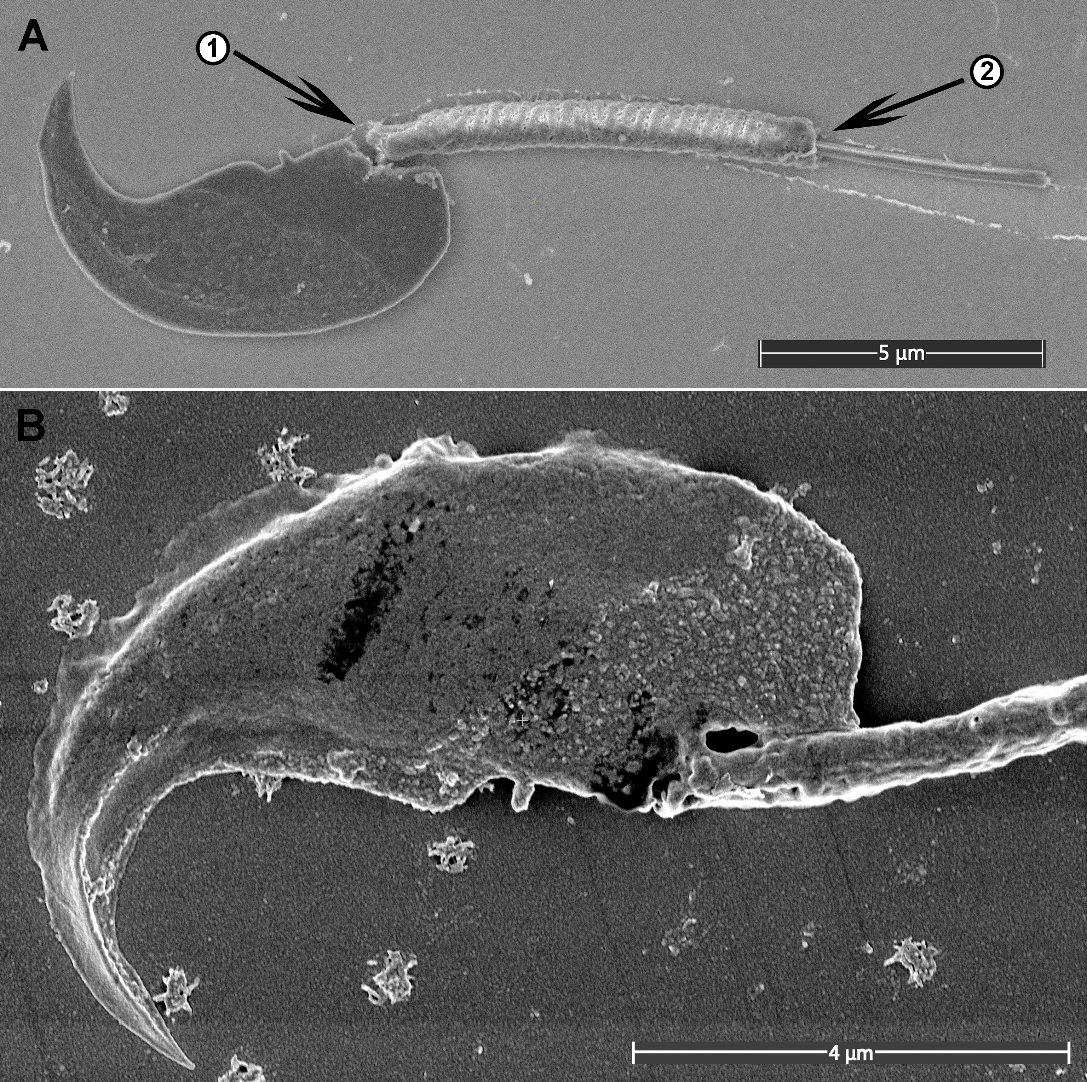


**Figure S1.** (A) Typical mechanical damages of sperm – rupture in the head-tail-coupling apparatus (1) and cell disaggregation (2). (B) Degradation of the cell surface with too long (4 h, room temperature) incubation in glutaraldehyde fixative.

**Figure S2.** Cell targeting and orientation of FIB milling planes. (A, B) Light microscopy visualisation of the top surface of the resin block with embedded monolayer of sperm through a layer of gold (A) and from the area where gold is removed (B). The speed/quality rate is depending on the milling planes orientation. To reach the best resolution the milling plane should be orthogonal to the cell axis (B, C, 1). To achieve the fastest data collection with reduced resolution the milling plate should be parallel to the cell axis (B, C, 2). S = sperm.
